# Recent advances in targeted therapy for Ewing sarcoma

**DOI:** 10.12688/f1000research.8631.1

**Published:** 2016-08-25

**Authors:** Kathleen I. Pishas, Stephen L. Lessnick

**Affiliations:** 1 Cancer Therapeutics Laboratory, Center for Personalized Cancer Medicine, Discipline of Medicine, University of Adelaide, Adelaide, SA, Australia; 2 Center for Childhood Cancer and Blood Disorders, The Research Institute at Nationwide Children's Hospital, Columbus, OH, USA; 3 Division of Pediatric Hematology/Oncology/Bone Marrow Transplant, Ohio State University, Columbus, OH, USA

**Keywords:** Ewing sarcoma, sarcoma, ganitumab, EWS/FLI

## Abstract

Ewing sarcoma is an aggressive, poorly differentiated neoplasm of solid bone that disproportionally afflicts the young. Despite intensive multi-modal therapy and valiant efforts, 70% of patients with relapsed and metastatic Ewing sarcoma will succumb to their disease. The persistent failure to improve overall survival for this subset of patients highlights the urgent need for rapid translation of novel therapeutic strategies. As Ewing sarcoma is associated with a paucity of mutations in readily targetable signal transduction pathways, targeting the key genetic aberration and master regulator of Ewing sarcoma, the EWS/ETS fusion, remains an important goal.

## Introduction

Despite our burgeoning knowledge of the molecular and pathognomonic foundation of Ewing sarcoma oncogenesis, improvement in the survival of patients with primary metastatic or relapsed disease remains obstinately poor, with long-term survival rates of less than 30% ^1^. Ewing sarcoma is an aggressive, rare bone malignancy that primarily afflicts young adolescents in the second decade of life. Approximately 20–25% of patients present with clinically detectable metastases at diagnosis ^2^, and those lacking overt disease likely harbor micro-metastases as evident by the high rate of relapse at distant sites following surgical resection. Although the advent of multi-agent adjuvant chemotherapy has achieved remarkable progress in the treatment of localized disease (65–75% 5-year survival rate), no standard therapy exists for second-line treatment of relapsed and refractory Ewing sarcoma, despite extensive protocol-driven clinical research evaluating dose intensification and schedule optimization.

Ewing sarcoma is an orphan cancer; its parental lineage is unknown and is frequently disputed still to this day ^3^. The oncogenic phenotype is primarily driven by one underlying prototypical chromosomal translocation, fusion of the *EWS* gene on chromosome 22q24 with one of five E-twenty-six (ETS) transcription factor gene family members ( *FLI* ^4^, *ERG* ^5^, *ETV1* ^6^, *E1AF* ^7, 8^, and *FEV* ^9^). Of the EWS/ETS translocations, 85% of Ewing’s tumors harbor the EWS/FLI reciprocal translocation t(11;22)(q24;q12), which links the strong transcriptional activation domain of the EWS protein to the ETS DNA-binding domain of the FLI protein ^10^. The resulting chimeric EWS/FLI fusion functions as a constitutively active transcription factor which regulates a myriad of genes required for the oncogenic behavior of Ewing sarcoma. EWS/FLI binds DNA at either ETS-like consensus purine-rich sites containing a core GGAA motif or repetitive GGAA-microsatellite elements embedded within promoter/enhancer regions of numerous target genes. Indeed, 40–50% of genomic EWS/FLI-binding sites are associated with GGAA-microsatellites ^11^.

As Ewing sarcoma possesses one of the lowest mutation rates among all cancers (0.15 mutations per megabase) ^12, 13^, the nefarious activity of EWS/FLI has long been considered the ideal therapeutic target. Although the t(11;22)(q24;q12) translocation was first described 33 years ago ^14^ and numerous studies have validated that the tumorigenic phenotype of Ewing sarcoma is dependent on this master regulator, EWS/FLI-targeted therapies are only now beginning to be clinically evaluated. Constraining the Achilles’ heel of Ewing sarcoma has proven to be extremely problematic. Fusion proteins are notoriously challenging targets because of their disordered protein nature and lack of intrinsic enzymatic activity.

The primary focus of this review is to highlight the recent advances and new therapeutic developments for this aggressive neoplasm, and the particular focus is on four classes of experimental agents: (i) targeted agents that disrupt the binding of EWS/FLI to key functional protein partners, (ii) agents that reverse the transcriptional signature of EWS/FLI, (iii) inhibitors of LSD1 (lysine-specific demethylase 1), and (iv) inhibitors of PARP-1 (poly ADP ribose polymerase-1).

## Targeting EWS/FLI: the untouchable Achilles’ heel of Ewing sarcoma

The EWS/FLI ** translocation primarily serves as the most reliable diagnostic marker and, in the majority of cases, sole genetic aberration that drives Ewing sarcoma oncogenesis. Despite this prime candidate vulnerability, clinical translation of therapeutic strategies directed toward eliminating or inactivating EWS/FLI has been largely unsuccessful. Since the cloning of the EWS/FLI translocation in 1992 ^4^, several studies have demonstrated the critical nature of EWS/FLI to maintain the oncogenic growth of Ewing sarcoma cells. Reduction of EWS/FLI fusion levels through anti-sense/small interfering RNA (siRNA) or oligodeoxynucleotides significantly impairs the proliferative, invasive, and tumorigenic phenotype of Ewing sarcoma both *in vitro* and *in vivo* ^15– 19^. ** However, owing to poor pharmacokinetic properties, these approaches are not currently clinically feasible *.* An inherent disadvantage of oncogenic transcription factors such as EWS/FLI in terms of “druggability” is their lack of intrinsic enzymatic activity. This, coupled with the disordered nature of the EWS/FLI protein (inability to form rigid three-dimensional structures under physiological conditions), which is due to low overall hydrophobicity, preludes standard structure-based small-molecule inhibitor design through crystallographic structural assessment ^20, 21^. As EWS/FLI requires disorder to achieve maximal transactivation of transcription and to facilitate the protein-protein complexes that lead to oncogenesis, directed small-molecule disruption of EWS/FLI from key functional protein partners or transcriptional complexes (or both) has gained considerable attention over the past decade.

**Figure 1.**
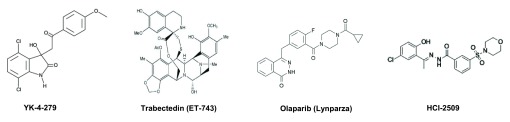
Chemical structures of Ewing sarcoma investigational agents.

To define proteins that directly interact and functionally modulate EWS/FLI, Toretsky *et al.* undertook phage library screening to identify peptides that could bind recombinant EWS/FLI ^22^. From the 28 peptides identified, EWS/FLI was shown to bind to the distal portion of the helicase domain of RNA helicase A (RHA) (K _D_ of 9.48 µM). Given that RHA enhanced EWS/FLI-modulated transcription, subsequent surface plasmon resonance screening of 3,000 compounds capable of binding monomeric EWS/FLI identified YK-4-279, a small molecule that could effectively dissociate EWS/FLI from RHA ( Figure 1). Owing to the chiral center of YK-4-279, comparisons of (S) and (R)-YK-4-279 enantiomer forms revealed that only racemic and the (S)-YK-4-279 enantiomer are able to block the interaction of EWS/FLI with RHA resulting in cell cytotoxicity and reduced EWS/FLI-driven transcriptional activation ^23^. Although Ewing sarcoma cell lines were highly sensitive to (S)-YK-4-279 compared with racemic, the short plasma half-life (0.585 and 0.583 hours, respectively) and low oral bioavailability of YK-4-279 could pose significant clinical challenges. Rates of absolute bioavailability of (S)-YK-4-279 following oral gavage in rats and intraperitoneal injection in mice were only 2–6% and 26%, respectively ^24^.

Although YK-4-279 was first thought to directly impede EWS/FLI-driven transcriptional activation, Selvanathan *et al.* recently demonstrated that the precise mechanism of action of YK-4-279 is through disruption of EWS/FLI protein interactions within the spliceosome leading to alternative splicing events that mirror EWS/FLI reduction ^25^. Indeed, initial Ewing sarcoma cell line viability assays demonstrated YK-4-279 anti-proliferative IC _50_ (half maximal inhibitory concentration) values of 0.5–2 µM, yet dissociation of EWS/FLI from RHA was achieved only following 10 μM YK-4-279 treatment ^22^. In verification of this new mechanism of action, YK-4-279 treatment did not mimic the transcriptional effects of EWS/FLI reduction, as only *VEGFA* and *TGFβR2* transcripts were altered in a fashion consistent with EWS/FLI reduction ^25^.

A near-universal problem in the era of targeted therapy is the emergence of primary or secondary drug resistance that permits tumor progression. Regardless of the route of YK-4-279 administration, sustained complete responses were not documented across a complete cohort of treated animals ^22, 24, 26^. As such, Lamhamedi-Cherradi *et al.* recently investigated both *de novo* and acquired mechanism(s) in which Ewing sarcoma cells evade YK-4-279-mediated apoptosis ^26^. YK-4-279 drug-resistant clones demonstrated enhanced proliferative rate over their parental cell lines and overexpressed c-Kit, cyclin D1, pStat3 (Y705) protein, and PKC isoforms β and δ. In contrast, pro-apoptotic proteins (such as Bim, Bax, Bid, and Bak) were significantly downregulated ^26^. In addition, YK-4-279 drug-resistant cells displayed significant cross-resistance to both the PKC inhibitor enzastaurin and the US Food and Drug Administration-approved c-Kit inhibitor imatinib.

YK-4-279 is the first EWS/FLI precision-guided drug candidate to show preclinical activity in Ewing sarcoma, and a phase 1 dose escalation study of intravenous TK216 (clinical derivative of YK-4-279) in patients with relapsed or refractory Ewing sarcoma (NCT02657005) is currently active for patient recruitment ( Table 1). The dosing schedule, administration route, and decision to use either racemic or (S)-YK-4-279 will have profound implications for clinical efficacy and resistance. It is with great anticipation that YK-4-279 can change the perceived dogma that transcription factors such as EWS/FLI are ubiquitously “undruggable”.

**Table 1.** Current Ewing sarcoma clinical trials.

Agent	Trial identifier	Sponsor	Phase	Age, years	Status
PARP inhibition					
Talazoparib and temozolomide	NCT02116777	NCI	I/II	1–30	Recruiting
Niraparib and temozolomide	NCT02044120	SARC	I	>13	Recruiting
Talazoparib (BMN-673)	NCT01286987	Medivation	I	>18	Active
Olaparib and temozolomide	NCT01858168	MGH	I	>18	Recruiting
Olaparib and trabectedin	NCT02398058	Italian Sarcoma Group	I	>18	Recruiting
Kinase inhibition					
Pazopanib	NCT01956669	Novartis/COG	II	1–18	Recruiting
Cabozantinib-s-malate	NCT02243605	NCI	II	>12	Recruiting
Regorafenib	NCT02389244	UniCancer	II	>18	Recruiting
Regorafenib	NCT02048371	SARC	II	>18	Recruiting
EWS/FLI inhibition					
TK216	NCT02657005	Tokalas	I	>12	Active
Miscellaneous					
Erlotinib in combination with temozolomide	NCT02689336	Washington University	II	1–21	Not Open
Abemaciclib (LY2835219) (CDK4/6 inhibition)	NCT02644460	Cynthia Wetmore	I	2–21	Recruiting
hu14.18K322A (anti-GD2 antibody)	NCT00743496	St. Jude Hospital	I	<21	Recruiting
Nivolumab with or without ipilimumab (IgG4 anti-PD-1 antibody)	NCT02304458	NCI	I/II	1–30	Recruiting
Lurbinectedin (PM01183)	NCT02454972	PharmaMar	II	>18	Recruiting
Linsitinib (anti-IGF-1R)	NCT02546544	University of Oxford	II	18–70 years	Recruiting

COG, Children’s Oncology Group; MGH, Massachusetts General Hospital; NCI, National Cancer Institute; PARP, poly ADP ribose polymerase; SARC, Sarcoma Alliance for Research through Collaboration.

## Reversing *EWS/FLI* gene signatures

The oncogenic phenotype of Ewing sarcoma is driven by the activating and repressive transcriptional functions of EWS/FLI ^27^. As such, therapeutic agents that can potentially reverse EWS/FLI-driven signatures and subsequently block the malignant proclivity of Ewing sarcoma have been an area of active interest by several groups. Trabectedin (ET-743, *Yondelis*), a synthetic alkaloid originally isolated from the marine ascidian *Ecteinascidia turbinata*, was recently shown to reverse the myxoid liposarcoma transcriptional program through DNA-binding inhibition of the oncogenic transcription factor FUS-CHOP ^28– 30^. It is proposed that trabectedin ( Figure 1) binds and alkylates DNA at the N2 position of guanine in the minor groove ^31^. Once bound, this reversible covalent adduct bends DNA toward the major groove, interferes directly with activated transcription, inhibits transcription-coupled nucleotide excision repair, promotes degradation of RNA polymerase II, and generates DNA double-strand breaks, leading to S and G _2_ cell cycle arrest ^32^. Grohar *et al.* demonstrated that Ewing sarcoma cell lines, in addition to myxoid liposarcomas, are particularly sensitive to the apoptotic effects of trabectedin compared with other fusion transcription factor-driven tumors, including embryonal/alveolar rhabdomyosarcoma and synovial sarcoma ^33^. Although protein levels of EWS/FLI remained unaffected following treatment, trabectedin reversed the EWS/FLI-induced gene expression signature, resulting in blockade of promoter activity and suppressed expression of critical EWS/FLI downstream targets such as NR0B1. Interestingly, of the four Ewing sarcoma cell lines tested, 5838 cells harboring the EWS/ERG translocation were the least sensitive. The authors suggest that this differential sensitivity may be attributed to the ERG transcription factor-binding domain which does not have a preferred trabectedin-binding site (CGG) overlapping its binding domain.

To develop trabectedin-based combination therapy with improved EWS/FLI suppression, Grohar *et al.* sought to identify genes driven by EWS/FLI that were suppressed following trabectedin treatment ^34^. A significant reduction in mRNA expression of the DNA damage response (DDR) REC Q helicase Werner syndrome protein (WRN) was observed following trabectedin treatment. Several studies have demonstrated that camptothecins can also directly suppress critical EWS/FLI downstream targets, including ID2 and NR0B1 ^35^. Since cells deficient in WRN are hypersensitive to the cytotoxic effects of camptothecins ^36, 37^, Grohar *et al.* investigated whether trabectedin could selectively sensitize Ewing sarcoma cells to the DNA-damaging effects of SN38 (active metabolite of irinotecan) ^34^. Compared with single-agent treatment *in vivo*, combinatorial treatment synergistically augmented the suppression of EWS/FLI targets, leading to enhanced formation of γH2AX foci (DNA double-strand breaks) and accumulation of cells in S phase. Furthermore, marked complete regression of xenograft tumors that persisted following withdrawal of treatment was observed with combination therapy that was more pronounced than treatment with either agent alone. In addition to camptothecins, insulin-like growth factor 1 receptor (IGF-1R) inhibitors (AVE1642 human antibody and linsitinib) significantly potentiated the efficacy of trabectedin both *in vitro* and *in vivo*. This highly synergistic cytotoxic combination was attributed to the ability of trabectedin to increase the occupancy of EWS/FLI to IGF-1R promoters, leading to IGF-1R upregulation. In contrast, binding of EWS/FLI (type I and type II) to the *TGFβR2* and *CD99* genes was strongly suppressed following both trabectedin and doxorubicin treatment ^38^. Indeed, EWS/FLI has been shown to directly affect IGF-1R signaling through suppression of IGFBP-3 (insulin-like growth factor-binding protein-3), leading to constitutive activation of the IGF-1 pathway ^39, 40^.

Despite the nanomolar sensitivity of Ewing sarcoma cells *in vitro*, trabectedin did not demonstrate sufficient single-agent activity in the recent Children’s Oncology Group (COG) phase II trial of trabectedin in children with recurrent Ewing sarcoma ^41^. Of the 10 evaluable patients with Ewing sarcoma, one patient achieved stable disease (15 cycles), and progressive disease was reported for the remaining nine patients. Grohar *et al.* hypothesized that these disappointing clinical results may be attributed to a narrow therapeutic index that limited or transiently achieved the required serum levels necessary to sufficiently inhibit EWS/FLI activity ^34^. Patient maximum concentration (C _max_) plasma levels of 2.49 ± 2.25 ng/ml (1.5 mg/m ^2^ dose) were attained and this was significantly lower than the 5–10 nmol/L trabectedin concentration required to suppress EWS/FLI downstream target expression *in vitro* ^34, 41^. In the initial phase I refractory solid tumor COG pharmacokinetic study of trabectedin, the only complete response documented was observed from a patient with Ewing sarcoma and was sustained for 10 months before recurrence 3 months after cessation of treatment ^42^. Unlike those in the phase II trial, patients in the phase I trial achieved C _max_ plasma levels of 10.52 ± 5.00 ng/ml (1.3 mg/m ^2^ dose). Although trabectedin in combination with olaparib is currently being assessed in a phase 1b clinical trial for patients with unresectable advanced/metastatic sarcomas (NCT02398058) ( Table 1), camptothecins should also be considered to suppress the EWS/FLI-mediated tumorigenic gene signature.

Recently, Hensel *et al.* demonstrated that EWS/FLI expression levels are significantly reduced in Ewing sarcoma cell lines following treatment with the BET (bromo and extraterminal) inhibitor JQ1 ^43^. It is proposed that JQ1 binds competitively to acetyl-lysine recognition motifs, thereby displacing bromodomain fusion oncoproteins such as BRD4 from chromatin ^44^. In addition to caspase-dependent apoptosis, microarray analysis of JQ1-treated Ewing sarcoma cell lines demonstrated significant downregulation of EWS/FLI transcriptional targets, including *DKK2*, *EZH2*, *GPR64*, *STEAP1*, and *STK32B*. This reversal of EWS/FLI gene expression pattern was exclusively mirrored through siRNA-mediated knockdown of BRD3 and BRD4 but not BRD2, suggesting that BRD3 and BRD4 may be critical epigenetic regulators in Ewing sarcoma. Although complete Ewing sarcoma xenograft tumor response was not observed following single-agent JQ1 treatment, these early preclinical findings suggest that combination treatment with epigenetic inhibitors that block BET bromodomain activity and the associated EWS/FLI transcriptional program may represent a potential therapeutic platform for Ewing sarcoma.

## Inhibitors of lysine-specific demethylase 1

Gene expression profiling and tissue microarray analysis of more than 500 sarcomas by Bennani-Baiti *et al.* revealed that the FAD monoamine oxidase LSD1 (lysine-specific demethylase 1, KDM1A) is highly overexpressed in Ewing sarcoma ^45^. Indeed, analysis of the Broad Institute Cell Line Encyclopedia ^46^ indicates that Ewing sarcoma is the second highest LSD1-expressing malignancy out of a comprehensive panel of 36 cancer subtypes. Previous mechanistic studies conducted by our laboratory suggested that the transcriptional repressive function of EWS/FLI is mediated through interaction with the NuRD co-repressor complex in which LSD1 functions as a key component ^27^. As such, the high expression of LSD1 in Ewing sarcoma, coupled with the absence of mutations ^47^ and its critical role in EWS/FLI transcriptional repression, provides a strong case for therapeutic intervention. Although the development of specific LSD1 inhibitors is still in its infancy, treatment of Ewing sarcoma cell lines with the specific and non-competitive reversible LSD1 inhibitor HCI-2509 ( Figure 1) comprehensively reversed the transcriptional signature driven by both EWS/FLI and EWS/ERG ^27, 48^. In addition, cells expressing EWS/FLI were approximately 10-fold more susceptible to the apoptotic cytotoxic effects of HCI-2509, underscoring the specificity of HCI-2509 for the treatment of Ewing sarcoma. Currently, three irreversible LSD1 inhibitory agents—tranylcypromine, GSK-2879552, and ORY-100—are undergoing clinical evaluation primarily in patients with acute myeloid leukemia ^47^, and clinical formulations of HCI-2509 are expected to enter phase I testing in 2017. Findings from these trials will guide the impetus for targeting LSD1-overexpressing cancers and have the potential to be a significant component in the armamentarium arsenal for the treatment of Ewing sarcoma.

## Inhibitors of poly ADP ribose polymerase

**Figure 2.**
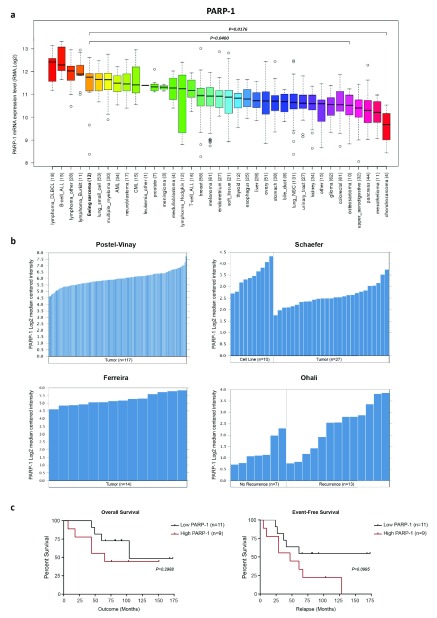
PARP-1 is highly expressed in Ewing sarcoma tumors and cell lines. ( **a**) Broad Institute PARP-1 expression across a panel of 1,036 cell lines. ( **b**) PARP-1 expression (microarray) in Ewing sarcoma tumors and cell lines. ( **c**) PARP-1 expression is not correlated with overall or event-free survival in Ewing sarcoma (n = 20). Survival data sourced from Ohali *et al.* ^51^ (2004). PARP-1, poly ADP ribose polymerase 1.

The PARP superfamily of multi-functional enzymes comprises 18 members, and PARP-1 is the most abundant (>85% of PARP activity) ^49^. The most well-established role of PARP-1 is the spatial and temporal organization of DNA single-strand break base excision repair with inhibition leading to stalled/collapsed replication forks and consequently catastrophic DNA double-strand breaks. Analysis of the Broad Institute Cell Line Encyclopedia ^46^ indicates that Ewing sarcoma is the fifth highest PARP-1-expressing malignancy and that expression is significantly higher than that of other solid bone sarcomas such as osteosarcoma ( *P* = 0.0400) and chondrosarcoma ( *P* = 0.0176) ( Figure 2a). Oncomine tumor microarray analysis also indicates that PARP-1 is highly expressed in Ewing tumors ^50– 53^ ( Figure 2b) but is not associated with overall or event-free survival, even though significantly higher expression was observed in relapsed patients ( *P* = 0.0252) ^51^ ( Figure 2b, c). In addition, mutations in PARP-1 and PARP-2 are seldom observed in Ewing sarcoma ( Table 2). Recent whole genome/exome sequencing studies of Ewing sarcoma tumors ^12, 13, 54– 56^ identified PARP-1/2 mutations in only 1 out of 279 (0.36%) and 2 out of 279 (0.72%) patient tumor samples, respectively. This empirical evidence, coupled with two landmark studies that highlighted the exquisite hypersensitivity of Ewing sarcoma cell lines to PARP inhibitors ^57, 58^, provided the premise for targeted PARP intervention for the treatment of Ewing sarcoma.

**Table 2.** Frequency of PARP-1 and PARP-2 mutations in Ewing sarcoma.

Study	Sequencing platform	Patient cohort	PARP-1 mutations	PARP-2 mutations
Crompton *et al.* ^13^	WES	n = 92	n = 1 (1.1%) (K203R)	n = 2 (E222Q, H428H)
Tirode *et al.* ^56^	WGS	n = 112	n = 0 (0%)	n = 0 (0%)
Agelopoulos *et al.* ^54^	WES	n = 50	n = 0 (0%)	n = 0 (0%)
Huether *et al.* ^55^	WGS	n = 19	n = 0 (0%)	n = 0 (0%)
Brohl *et al.* ^12^	WGS	n = 6	n = 0 (0%)	n = 0 (0%)
Total			n = 1/279 (0.36%)	n = 2/279 (0.72%)

PARP, poly ADP ribose polymerase; WES, whole exome sequencing; WGS, whole genome sequencing.

Comprehensive drug screening (130 compounds) across 639 human tumor cell lines by Garnett *et al.* ^57^ identified a highly significant association between the presence of the EWS/FLI rearrangement and olaparib (Lynparza) sensitivity (geometric mean IC _50_ for EWS/FLI = 4.7 versus 64 μM for non-EWS/FLI lines). Indeed, sensitivity of Ewing sarcoma cell lines to both olaparib and the structurally distinct PARP inhibitor AG-014699 was comparable to that observed in BRCA-deficient cell lines and greater than that observed from other solid bone and soft tissue sarcomas. Mechanistic investigations by Brenner *et al.* validated that the marked sensitivity of Ewing sarcoma cell lines to olaparib could be attributed to a positive feedback loop in which the EWS/FLI fusion drives and maintains PARP-1 expression, which in turn further promotes transcriptional activation by EWS/FLI ^58^. Knockdown of EWS/FLI in Ewing sarcoma cells led to a significant reduction in both PARP-1 protein expression and promoter activity. Surprisingly, marked differences in single-agent cytotoxicity across several PARP inhibitors (talazoparib, niraparib, olaparib, and veliparib) has been documented, and talazoparib (BMN-673) and veliparib are the most and least active compounds in Ewing sarcoma, respectively ^59– 61^. In addition to catalytic inhibition, PARP inhibitors exert their cytotoxicity by tightly trapping PARP-1 and PARP-2 to DNA at sites of single-strand breaks, and *in vitro* Ewing sarcoma inhibitor sensitivity correlates with PARP trapping potential ^60, 62^. Whole exome sequencing of Ewing sarcoma cell lines revealed an absence of mutations in DNA repair genes, and as both ATM and ATR DDR signaling pathways remain functional in Ewing sarcoma, PARP inhibitor sensitivity is not underpinned by mutational defects in DNA repair by homologous recombination but perhaps through hypersensitivity to trapped PARP-1 DNA complexes ^61^.

Despite the acute hypersensitivity of Ewing sarcoma cell lines to numerous PARP inhibitors *in vitro*, these results did not translate directly to single-agent xenograft responses *in vivo* ^58, 60, 63– 65^. Olaparib monotherapy only led to a significant delay in Ewing sarcoma xenograft models. However, combined treatment with the DNA-alkylating agent temozolomide resulted in sustained complete responses without observable recurrence ^58^. Consistent with the minimal activity of single-agent olaparib in Ewing sarcoma xenografts, no objective responses (partial or complete) were observed from the first phase II study of olaparib (NCT01583543) in 12 patients with refractory Ewing sarcoma, median time to progression was 5.7 weeks ^66^, thus underscoring the requirement for combination therapies. To investigate the ability of PARP inhibitors to modulate the chemosensitivity of Ewing sarcoma, Engert *et al.* screened PARP synergistic drug interactions with Ewing sarcoma chemotherapeutic backbone cassettes ^59^. Indeed, the strongest synergism was observed in combination with temozolomide followed by SN38, with diminutive synergistic effects observed with actinomycin D and vincristine. Of note, triple therapy comprising olaparib, temozolomide, and SN38 significantly reduced the viability of Ewing sarcoma cells compared with single-agent or co-treatment with temozolomide or SN38 *in vitro*. Consistent with these findings, no significant difference was observed in overall survival in a phase III preclinical orthotropic mouse model of Ewing sarcoma treated with placebo or single-agent PARP inhibitors veliparib, olaparib, and talazoparib ^60^. However, combination of PARP inhibitors (olaparib or BMN-673) with temozolomide and irinotecan gave complete and durable responses in 71–88% of mice respectively.

Inhibitors of PARP have emerged as a novel class of agents to treat Ewing sarcoma, and several clinical studies are underway ( Table 1). It is clear that single-agent PARP treatment is ineffective and will require strategic combinatorial strategies with temozolomide, topoisomerase I poisons, or radiotherapy to achieve maximal therapeutic effect. Results from these ongoing adult studies are eagerly awaited to help guide future trials for pediatric and adolescent patients with Ewing sarcoma.

## Conclusions

Though Ewing sarcoma was first described 95 years ago, its treatment still relies on conventional multi-agent chemotherapeutic regiments in combination with surgery or radiotherapy or both. This current backbone is associated with considerable acute and long-term toxicities, and since further modification of the five-drug chemotherapeutic cassette seems unlikely to produce additional benefits, successful integration of novel targeted agents is urgently required in order to improve outcomes for patients with relapsed and metastatic disease. The first attempt of targeted therapy integration is currently being undertaken in a randomized phase II trial evaluating the addition of ganitumab (IGF-1R human monoclonal antibody) to multi-agent chemotherapy (vincristine, doxorubicin, cyclophosphamide, ifosfamide, and etoposide) for patients with newly diagnosed metastatic Ewing sarcoma (AEWS1221/NCT02306161). It is hoped that combining IGF-1R targeted agents with conventional therapy may lower the effective dosage of radiotherapy and chemotherapy in addition to minimizing side effects while maintaining efficacy.

Metastatic disease at the time of presentation or at relapse remains the single most powerful predictor of outcome in Ewing sarcoma, and the mechanisms that drive metastasis remain largely unknown. Whether targeted agents that directly inhibit critical EWS/FLI protein-protein interactions or reverse the EWS/FLI transcriptional signature can successfully prevent or delay tumor progression remains as yet unanswered and will be the focus of the next generation of phase I/II trials, whose ultimate goal is to determine whether these novel therapies can significantly improve survival outcomes for patients with Ewing sarcoma.
